# Peripheral Ion Channel Gene Screening in Painful- and Painless-Diabetic Neuropathy

**DOI:** 10.3390/ijms23137190

**Published:** 2022-06-28

**Authors:** Milena Ślęczkowska, Rowida Almomani, Margherita Marchi, Bianca T. A. de Greef, Maurice Sopacua, Janneke G. J. Hoeijmakers, Patrick Lindsey, Erika Salvi, Gidon J. Bönhof, Dan Ziegler, Rayaz A. Malik, Stephen G. Waxman, Giuseppe Lauria, Catharina G. Faber, Hubert J. M. Smeets, Monique M. Gerrits

**Affiliations:** 1 Department of Toxicogenomics, Maastricht University, 6229 ER Maastricht, The Netherlands; rfalmomani7@just.edu.jo (R.A.); patrick.lindsey@maastrichtuniversity.nl (P.L.); bert.smeets@maastrichtuniversity.nl (H.J.M.S.); 2 School of Mental Health and Neuroscience, Maastricht University, 6229 ER Maastricht, The Netherlands; 3 Department of Neurology, Maastricht University Medical Centre, 6229 HX Maastricht, The Netherlands; bianca.greef@mumc.nl (B.T.A.d.G.); maurice.sopacua@mumc.nl (M.S.); j.hoeijmakers@mumc.nl (J.G.J.H.); c.faber@mumc.nl (C.G.F.); 4 Department of Medical Laboratory Sciences, Jordan University of Science and Technology, Irbid 22110, Jordan; 5 Neuroalgology Unit, IRCCS Foundation “Carlo Besta” Neurological Institute, 20133 Milan, Italy; margherita.marchi@istituto-besta.it (M.M.); erika.salvi@istituto-besta.it (E.S.); giuseppe.lauriapinter@istituto-besta.it (G.L.); 6 Institute for Clinical Diabetology, German Diabetes Center, Leibniz Center for Diabetes Research at Heinrich Heine University Düsseldorf, 40225 Düsseldorf, Germany; gidon.boenhof@ddz.uni-duesseldorf.de (G.J.B.); dan.ziegler@ddz.de (D.Z.); 7 Department of Endocrinology and Diabetology, Medical Faculty, University Hospital Düsseldorf, Heinrich Heine University Düsseldorf, 40225 Düsseldorf, Germany; 8 Division of Cardiovascular Sciences, University of Manchester, Manchester M13 9PL, UK; ram2045@qatar-med.cornell.edu; 9 Department of Medicine, Weill Cornell Medicine-Qatar, Doha P.O. Box 24144, Qatar; 10 Department of Neurology and Center for Neuroscience and Regeneration Research, Yale University School of Medicine, New Haven, CT 06510, USA; stephen.waxman@yale.edu; 11 Center for Neuroscience and Regeneration Research, Veterans Affairs Medical Center, West Haven, CT 06516, USA; 12 Department of Clinical Genetics, Maastricht University Medical Centre, 6229 HX Maastricht, The Netherlands; monique.gerrits@mumc.nl

**Keywords:** MIPs-NGS, neuropathic pain, ion channel, TRP channels, diabetic neuropathy

## Abstract

Neuropathic pain is common in diabetic peripheral neuropathy (DN), probably caused by pathogenic ion channel gene variants. Therefore, we performed molecular inversion probes-next generation sequencing of 5 transient receptor potential cation channels, 8 potassium channels and 2 calcium-activated chloride channel genes in 222 painful- and 304 painless-DN patients. Twelve painful-DN (5.4%) patients showed potentially pathogenic variants (five nonsense/frameshift, seven missense, one out-of-frame deletion) in ANO3 (*n* = 3), HCN1 (*n* = 1), KCNK18 (*n* = 2), TRPA1 (*n* = 3), TRPM8 (*n* = 3) and TRPV4 (*n* = 1) and fourteen painless-DN patients (4.6%—three nonsense/frameshift, nine missense, one out-of-frame deletion) in ANO1 (*n* = 1), KCNK18 (*n* = 3), KCNQ3 (*n* = 1), TRPA1 (*n* = 2), TRPM8 (*n* = 1), TRPV1 (*n* = 3) and TRPV4 (*n* = 3). Missense variants were present in both conditions, presumably with loss- or gain-of-functions. KCNK18 nonsense/frameshift variants were found in painless/painful-DN, making a causal role in pain less likely. Surprisingly, premature stop-codons with likely nonsense-mediated RNA-decay were more frequent in painful-DN. Although limited in number, painful-DN patients with ion channel gene variants reported higher maximal pain during the night and day. Moreover, painful-DN patients with TRP variants had abnormal thermal thresholds and more severe pain during the night and day. Our results suggest a role of ion channel gene variants in neuropathic pain, but functional validation is required.

## 1. Introduction

Neuropathic pain (NeuP) is caused by damage or disease of the somatosensory nervous system [1]. Around 50% of patients with diabetes develop diabetic peripheral neuropathy (DN) [2]. NeuP impacts quality of life negatively in affected individuals and is associated with anxiety and depression [1]. Unfortunately, available treatment for neuropathic pain is largely unsatisfying, with less than 50% of patients achieving 50% pain relief [3].

Several small-scale studies have investigated why some patients with diabetic neuropathy develop painful-DN, while others do not [4]. Female sex, longer diabetes duration, older age, higher body mass index and smoking have been reported as risk factors [5,6,7]. However, these results were not confirmed in large cross-sectional studies performed in patients with type 2 diabetes, except for the association with smoking [8]. The complexity of the condition, limited number of participants, different assessment methods and definition of DN make it difficult to conclude if painful-DN and painless-DN represent different disorders or are different manifestations of the same disease [3,4].

In recent years, genetic causes of NeuP have partially been resolved, and next-generation sequencing (NGS) has been increasingly performed to identify genes and genetic variants associated with NeuP [6]. Pathogenic variants have been identified in voltage-gated sodium ion channels (VGSCs), especially the α-subunits. Voltage-gated potassium channels are a large group of channels, opening in response to membrane depolarization [9]. VGSCs are transmembrane proteins expressed in the central and peripheral nervous system that play an important role in cells’ electrical signaling [9]. The loss-of-function mutation in the *SCN9A* gene encoding Na_v_1.7, in contrast, led to congenital insensitivity to pain, while gain-of-function mutations have been linked to a spectrum of pain disorders, including painful-DN [9]. Patients with painful-DN and rare Na_v_1.7 variants report more severe burning pain and increased pressure stimuli sensitivity, compared to painful- DN patients without the *SCN9A* variant [10,11]. However, the majority of genetic factors contributing to painful-DN still remain to be identified.

Apart from VGSCs, other ion channels are also essential for proper functioning of the nervous system [11]. It has been reported that transient receptor potential (TRP) cation channels, voltage-gated potassium (Kv) channels and hyperpolarization-activated and cyclic nucleotide-gated channels (HCN) may play a role in pain modulation and processing and/or painful neuropathies [11,12,13,14]. TRP channels are responsible for thermal, chemical and mechanical sensations [15]. HCN channels contribute to electrical excitability and pace-making activity in neuronal cells and have been linked to neuropathic pain [11,13]. In addition, anoctamins have been reported in pain processing and modulation of neuropathic pain [16,17]. ANO1 is the most studied Ca^2+^-activated Cl- channel from the ANO family, which interacts with TRPV1, leading to pain enhancement in sensory neurons, while ANO3 regulates pain processing via increasing the Slack channels’ activity in DRG neurons [16,17,18].

Our hypothesis is that in addition to VGSCs, variants in other ion channels are involved in painful-DN. Therefore, we analyzed seven Kv, five TRP, two ANO and one HCN ion channel genes expressed in peripheral nerves, using single molecule molecular inversion probes-next generation sequencing (smMIPs-NGS) in patients with painful-DN and painless-DN. The painless-DN group was included to identify variants linked to the absence of pain, which may play role in pain resilience and to exclude common variants present frequently in both patient groups. We also assessed if these variants were linked to specific clinical manifestations.

## 2. Results

### 2.1. Patient Characteristics

In total, 612 patients with DN were included, 393 from the Heinrich Heine University (Düsseldorf, Germany), and 219 from the University of Manchester (Manchester, UK). Of these, 86 patients were excluded, due to low DNA quality, MIP failure, incomplete clinical data or withdrawal of formal consent. Among the enrolled patients, 222 had painful-DN and 304 had painless-DN. The mean age at recruitment was 64.5 years old (SD +/−11.2 years). Sex distribution in the study group was 372 (70.7%) males versus 154 (29.3%) females. The higher male prevalence was present in both phenotypes. Clinical characteristics of the study population are presented in Table S2.

### 2.2. Performance of smMIPs-NGS

To assess the performance, capture efficiency and coverage of exons and exon-flanking sequences (+/−20 bps) of 15 ICG, data of 50 samples from 5 different NGS runs were used. An overall coverage of more than 30×/bp was obtained for 93% of the on-target regions. Exons and exon-flanking sequences of ten gene areas have an average coverage of >90%. Four exons had poor coverage (<20×/bp) or were completely missing (exon 1, exon 12 and exon 20 of *ANO1*, and exon 3 of *TRPV1*), and MIPs for 14 exons covered the targeted region of > 20×/bp only partially (Table S3). For the remaining exons and exon-flanking sequences, the average coverage was <90%, but at least an average coverage of 84% was reached.

### 2.3. Genetic Screening of 15 Ion Channels in Painful-Diabetic Neuropathy

**Figure 1:**
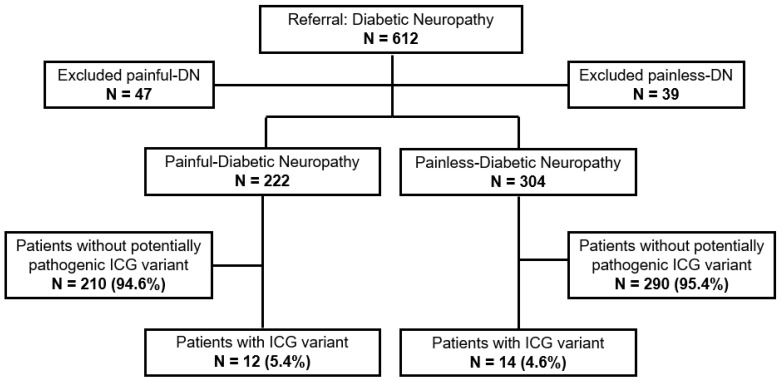
Patients with painful- and painless-diabetic neuropathy screened for potentially pathogenic variants in 15 ion channel gene panel. ICG, Ion Channel Gene; DN, Diabetic Peripheral Neuropathy; N, number.

MIP-NGS data analysis of 222 patients with painful-DN resulted in the identification of 13 different potentially pathogenic variants in 6 ICG genes (*ANO3*, *HCN1*, *KCNK18*, *TRPA1*, *TRPM8*, *TRPV4*, Table 1). Eleven patients were positive for one heterozygous variant, and one patient harbored two heterozygous variants (*n* = 12/222, 5.4%, Figure 1). All 13 variants were classified as variants of uncertain clinical significance (VUS, Table 1). Nine out of thirteen identified ICG variants were novel (not reported in the literature, the Human Gene Mutation Database (HGMD) or ClinVar). Two frameshift mutations localized in the *KCNK18* gene, c.414_415del and c.361dup, have been functionally linked to migraines [19,20,21]. The *ANO3* missense variant c.638C>T was reported in ClinVar as VUS without phenotype and *TRPV4* c.2336+1G>A as VUS for Charcot-Marie-Tooth disease. Most of the variants were located in genes from the TRP family, responsible for mechanical, chemical and thermal sensations. The painful-DN patients with an ICG variant have been previously tested for variants in *SCN3A*, *SCN7A-SCN11A* and *SCN1B-SCN4B*. None of them harbored a VUS, a likely pathogenic or pathogenic variant in one of these genes (unpublished data). The clinical characteristics of painful-DN patients with ICG VUS are presented in Table 2 and the autonomic complaints are summarized in Supplementary Materials (Table S5, supplementary data). Most of the patients (seven out of nine; for three patients, TTT is missing) with potentially causative ICG variant had an abnormal TTT, especially patients carrying variants in one of TRP genes (four out of four; for two patients, TTT is missing). The majority of patients with painful-DN with an ICG variant reported a negative family history for neuropathy and 6 out of 12 patients had identified a possible non-genetic underlying cause (Table 2). Patients with painful-DN with identified ICG variants (*n* = 9) reported higher mean maximal pain scores during 24 h (PI-NRS 7.2 +/−2.4), compared to patients with painful-DN without an ICG variant (PI-NRS 6.0 +/−3.0, *n* = 150). However, due to the small sample size, statistical analysis was not possible (Table S4). Interestingly, a patient with painful-DN and a potentially causative variant in the TRP gene had severe or very severe pain during night (PI-NRS 8.5 +/−1.29) and day (PI-NRS 8.0 +/−0.82) (Table 2) and impaired thermal sensation.

### 2.4. Genetic Screening of 15 Ion Channels in Painless Diabetic Neuropathy

In 304 patients with painless-DN, 13 different potentially pathogenic heterozygous variants were identified. Fourteen (4.6%) patients had a potentially pathogenic variant in one of the fifteen ICG, but none had more than one ICG variant (Table 3). In two individuals with an ICG variant, previously, a potentially pathogenic SCN variant was reported; *SCN10A* c.368C>T, VUS and *ANO1* c.1892G>A, VUS, and *SCN10A* c.1141A>G, possibly pathogenic and *TRPV4* c.958C>T, VUS. Potentially pathogenic ICG variants were located in the following seven genes: *ANO1*, *KCNK18*, *KCNQ3*, *TRPA1*, *TRPM8*, *TRPV1* and *TRPV4* (Table 3). Seven out of thirteen detected variants were novel. Six variants have been reported in ClinVar as pathogenic, VUS or variant with conflicting interpretations of pathogenicity. Both *KCNK18* variants have been linked to migraines and reported respectively as pathogenic, but with conflicting interpretations of pathogenicity. *KCNQ3* c.1226C>G was reported as VUS in benign familial neonatal seizures. *TRPV4* c.711A>G and c.1039G>T were reported as VUS for Charcot-Marie-Tooth disease type 2C and *TRPV4* c.958C>T as a variant with conflicting interpretations of pathogenicity for skeletal dysplasias, spinal muscular atrophies and Charcot-Marie-Tooth. Five painless-DN patients with an ICG variant filled out a pain questionnaire and all of them reported no pain during 24 h; max and mean pain during night and day (PI-NRS 0). It seems that patients with painless-DN with an ICG VUS variant reported less often autonomic complains, especially diarrhea, dry mouth, orthostatic dizziness, sheet intolerance and restless leg; however, the group size (*n* = 4) is too small to draw a definite conclusion. The clinical characteristic and autonomic complaints in patients with painless- DN and ICG variant are presented in Table 4 and Table S6 in Supplementary Materials.

## 3. Discussion

### 3.1. Summary of Detected Variants

In our cohort of patients with DN, twelve patients with painful-DN (5.4%) and fourteen patients with painless-DN (4.6%) harbored at least one potentially pathogenic variant in the ICG gene, respectively linked to pain or no pain. Only one patient with painful-DN had two heterozygous variants TRPA1 c.2481del and TRPM8 c.1437G>T. In total, we identified 13 different potentially pathogenic variants in painful-DN located in *ANO3*, *HCN1*, *KCNK18*, *TRPA1*, *TRPM8*, *TRPV4*. Three patients (1.4%) had a potentially pathogenic variant in *ANO3*, one (0.5%) in *HCN1*, two (0.9%) in *KCNK18*, three (1.4%) in *TRPA1*, three (1.4%) in *TRPM8*, one (0.5%) in *TRPV4*. In patients with SFN and NeP, the frequency of (potentially) pathogenic variants in the sodium ion channels was slightly higher; 5.1% for *SCN9A*, 3.7% for *SCN10A* and 2.9% for *SCN11A* [24]. A total of 13 different potentially pathogenic variants was detected in painless-DN in *ANO1*, *KCNK18*, *KCNQ3*, *TRPA1*, *TRPM8*, *TRPV1* and *TRPV4.* Potentially causative variants were identified in one patient (0.3%) in *ANO1*, three (1%) in *KCNK18*, one (0.3%) in *KCNQ3*, two (0.7%) in *TRPA1*, one (0.3%) in *TRPM8*, three (1%) in *TRPV1* and three (1%) in *TRPV4*. Four of these genes (*ANO1*, *ANO3*, *HCN1* and *KCNQ3*) have not been linked to a painful phenotype or to pain insensitivity before in HGMD. We did not find any potentially pathogenic variant in the following six genes from our gene panel: *KCNA2*, *KCNA4*, *KCNNA*, *KCNQ5*, *KCNS1*, *TRPV3*.

The same missense variants were not present in any of the ICG genes in painful- and painless-DN, with the exception of migraine-related *KCNK18* variants c.414_415del and c.361dup and *TRPA1* c.352C>G. Our patients did not report migraines during clinical investigation. However, questions about headaches or migraines were not included in the standard questionnaires and we were not able to contact the patients to confirm the lack of migraine symptoms. The presence of these variants in both phenotypes might be explained by other mutations in the genes not included in our gene panel or non-genetic risk factors contributing to a painful phenotype [6,25]. In two individuals with an ICG variant, previously, a potentially pathogenic SCN variant was reported; *SCN10A* c.368C>T, VUS and *ANO1* c.1892G>A, VUS, and *SCN10A* c.1141A>G, possibly pathogenic and *TRPV4* c.958C>T, VUS.

### 3.2. Effect of VUS on Protein Function

All variants meeting our criteria were variants of uncertain clinical significance, making it difficult to draw a definite conclusion on a causative role. However, this is not surprising, since most of the variants we identified were novel and we could only rely on in-silico predictions without segregation analysis in families or functional studies. The development of NGS and more affordable cost of sequencing have resulted in recent years in the identification of a significant number of VUS and part of them have been proved to be pathogenic over the time [26].

The VUS detected in our study either change the protein via substitutions of conserved amino acids (missense VUS) or are generally predicted to lead to the absence of the protein, because of nonsense mediated decay, triggered by a premature stop codon (nonsense/frameshift VUS). For the latter group, the KCNK18 gene contained premature stop codons in both painful- and painless-DN, making a causal role of haploinsufficiency of these genes on the pain phenotype unlikely. For the other nonsense/frameshift variants, this is not the case. Although NMD is most likely, truncated non-functional protein or alternative processes, such as frameshift mutation-induced alternative translation initiation (fsATI), have also been reported [19], requiring functional validation of the frameshift/nonsense mutation to be sure of the mechanism.

Overlap exists between the genes with missense mutations in painful- and painless-DN, but this does not disqualify the role they might have, as it has been reported that missense mutations can lead to gain or loss-of-functions with possible opposite effects on pain sensitivity, highlighting the importance of functional effect rather than the variation itself. Additional support for functional relevance is for the 3 VUS in TRPV1 and 1 VUS in ANO3, located in the transmembrane domain, which can directly affect channel properties e.g., channel gating [27]. Two missense VUS were located in ankyrin repeat II-containing domain, responsible for protein-protein interactions and protein folding [28]. Dysfunction in ANK repeat proteins has been reported in many human diseases [28]. Seven missense VUS were located in the N- and the C-terminal part of the protein, which play an important role in protein stability and turnover [29]. Two missense VUS were found in intracellular linkers between transmembrane domains in TRPM8 and ANO1. Interestingly, most of the potentially pathogenic SCN9A variants were localized in the intracellular linkers [24]. Therefore, the VUS identified are expected to have a negative quantitative effect on protein function (nonsense/frameshift) or a GOF or LOF (missense), although the direction or magnitude cannot be predicted by bioinformatic tools.

### 3.3. Possible Link between VUS and Clinical Manifestations

#### 3.3.1. Ca^2+^ Channels

Three missense heterozygous variants in the *ANO3* gene were detected in painful-DN and one missense heterozygous variant in the *ANO1* gene in painless-DN. These genes belong to Ca^2+^-activated Cl- channels and they are highly expressed in dorsal root ganglia (DRG) [17,18]. ANO3 is known to regulate pain processing via interaction with Slack channels and it has been shown that ANO3 knockdown leads to reduced mechanical allodynia and pain attenuation [18]. Therefore, GOF ANO3 variants potentially increase protein activity, causing enhanced pain sensitivity in painful-DN. ANO1 has been described as a heat sensor and a knockout of the ANO1 gene reduces thermal nociceptive responses [17,30]. A LOF of the ANO1 VUS would confirm a role in the painless-DN phenotype.

#### 3.3.2. K^+^ Channels

Voltage-dependent potassium channels are crucial for controlling the excitability of nociceptors and pain processing [31]. A heterozygous missense variant HCN1 c.1214G>A was identified in a patient with painful-DN. It has been reported that both inflammatory and neuropathic pain are rapidly inhibited by blocking HCN-dependent repetitive firing in peripheral nociceptive neurons [32,33,34]. Furthermore, the specific blockade of HCN1 attenuates hyperalgesia and allodynia in a neuropathic pain animal model [35]. If the HCN1 VUS increases HCN-dependent firing, this would explain the opposite effect. Recent studies have demonstrated that the gain of function mutations of KCNQ2/3 channels contributes to pain resilience [36,37], which might explain the KCNQ3 c.1226C>G VUS variant present in a patient with painless-DN.

#### 3.3.3. TRP Channels

We identified several variants located in TRP channels both in painful- and painless- DN. TRPV1, TRPM8, and TRPA1 are thermal and chemical detectors that activate sensory neurons to produce pain, while TRPV4 mediates nociceptive behaviors by hyper- and hypotonic stimuli [38]. Three TRPA1 VUS variants were detected in painful-DN, including one missense c.352C>G and two variants (c.2481del and c.1954C>T), leading to a premature stop codon and most likely nonsense-mediated decay, predicted specifically in the case of the c.1954C>T variant. This shows that TRPA1 loss of function is linked to increased pain sensitivity. Moreover, the c.1954C>T was also detected in a patient with SFN and burning pain in the hands and lower legs (unpublished data). Interestingly, the TRPA1 c.2755C>T variant leading to a premature stop codon at position 919 has been found to co-segregate with pain in cram-fasciculation syndrome [39]. As for the mechanism, a toxic gain-of-function was proposed, which was consistent with a marked clinical improvement with carbamazepine, a cation (sodium) channel blocker. However, this would require expression of the truncated protein, for which no evidence was provided. Increased TRPA1 expression in DRG has been observed after nerve injury and has been linked to cold hyperalgesia, which was reversed after TRPA1 blockade [40]. Consistent with this, GOF mutation Asn855Ser caused cold hyperalgesia in familial episodic pain syndrome [41]. Two out of three painful-DN patients with a TRPA1 VUS (one missense, one nonsense) had an abnormal TTT; for one patient, the data were incomplete. In painless-DN, one missense TRPA1 VUS out of two has an abnormal TTT. This suggests a role of TRPA1 variants in thermal sensation; however, based on the limited number of samples, it is not possible to draw a definite conclusion, especially as abnormal thermal sensation is common in patients with diabetic neuropathy.

Similar to TRPA1, TRPM8 modulates the cold sensation and its activation produces analgesia in chronic neuropathic pain states [42,43]. In painful-DN, we detected one frameshift mutation, leading to a pre-mature stop codon and two missense TRMP8 VUS and two patients had abnormal TTT values. One TRPM8 VUS c.2956G>A was present in a patient with painless-DN and abnormal TTT, again stressing that for the missense mutations, GOF or LOF have to be defined first before drawing conclusions.

TRPV1 and TRPV4 belong to the transient receptor potential vanilloid family and most of the VUS in these genes were present in painless-DN. In total, three missense VUS in TRPV1 were present in painless-DN, all located in the functional domain of the channel; c.1450G>C in transmembrane domain II, c.1781C>T and c.1790C>T both in transmembrane domain IV. Activation of TRPV1 results in the generation of action potentials and in many cases, pain [44]. Moreover, the GOF TRPV1 mutation at position p.Q85R has been identified in three cases with persistent neuropathic corneal pain after refractive surgery [45]; therefore, variants reducing TRPV1 activity are expected in the painless-DN group. TRPV4 is involved in mechanical hyperalgesia and leads to pain behaviors in the animal model in response to hypo-osmotic stimulation and inflammation [46]. As indicated above, TRPV4 VUS variants leading to premature stop codons and most likely NMD were found in painless-DN. The splice-site variant c.2336+1G>A, r.spl? detected in painful-DN is predicted to skip exon 14, leading to a frameshift and premature stop codon (Table 1), whereas splice-site variant c.711A>G, r.spl? detected in pain-less DN most likely resulted in incorporation of intronic sequences in all cases, leading to a frame-shift and premature stop codon as well. Therefore, both variants can also be considered LOF function mutations, which would preclude a role in the pain phenotype. Evidently, confirmation at the mRNA or protein level is required before drawing a definite conclusion.

### 3.4. Painful-DN Patients with ICG Variant Have More Pain Than Painful-DN without ICG Variant

Patients with painful-DN with an ICG VUS (*n* = 9) had higher mean pain scores compared to patients with painful-DN without the potentially causative ICG variant (*n* = 150). Determining clinically important differences in pain is challenging [47], but applying a pain classification proposed by Serlin et al., where PI-NRS 0–4 is considered as mild pain, 5–6 moderate and 7–10 severe [48], we found that patients harboring an ICG variant had severe maximal pain during the day and night, while patients without an ICG variant reported moderate pain. Moreover, four patients with painful-DN with a TRP variant had severe max pain during night (PI-NRS 8.5 +/−1.29) and day (PI-NRS 8.0 +/−0.82), together with abnormal TTT. These observations have been made in our DN patient population, which includes more male than female patients; therefore, they might be less relevant for female patients. Confirmation in a cohort of female patients should definitely be considered.

### 3.5. Conclusions and Future Perspectives

Painful neuropathy is a heterogeneous, multifactorial disease with many potential genetic contributors and risk factors [25]. According to Eijkenboom et al., screening of SCN9A, SCN10A and SCN11A may reveal an underlying genetic cause in 11.6% of pure SFN patients, comparable with previous studies [24]. The same set of genes was used for screening painful- and painless-idiopathic peripheral neuropathy and diabetic polyneuropathy, identifying of low-frequency nonsynonymous missense variants in at least 1 of the 3 genes in 47.7% of patients [49]. Interestingly, there were no significant differences in the missense variant allele frequencies of SCN9A, SCN10A and SCN11A between patients with painful- or painless-peripheral neuropathy [49]. This overlap corroborates our findings in 15 other ICG in this group of patients. In a well characterized cohort of patients with diabetic neuropathy, we have identified potentially pathogenic variants in 5.4% of the patients with painful-DN and 4.6% of the patients with painless-DN. Co-segregation analysis or functional analysis was not possible, limiting pathogenicity scores to predominantly VUS. However, our results suggest that other ion channels, including TRPs, can be genetic contributors to neuropathic pain, making them interesting candidates for further investigation and potential targets for pain therapy.

## 4. Methods

### 4.1. Study Population and Clinical Assessment

Patients with painful-DN and painless-DN were enrolled in the Deutsche Diabetes Forschungsgesellschaft (DDFG), Heinrich Heine University, Düsseldorf, Germany or at the University of Manchester, Manchester, United Kingdom. They were examined between June 2014 and September 2016. Only patients aged above 18 years old and those diagnosed with diabetes mellitus type 1 or type 2 (World Health Organization criteria) were eligible for this study. Demographic data, medical history, age of onset of complaints, duration of symptoms, altered pain sensation and family history were recorded. Pain intensity was evaluated using the 11-point Numerical Rating Scale (PI-NRS) from 0–10, where 0 means “no pain” and 10 refers to “the worst pain imaginable.” For all the patients, informed consent was obtained and a blood sample for genetic analysis was drawn.

For the patients with painful-DN, quantitative sensory testing of the lower limb was performed on the dorsum of the foot using the Medoc^®^ device. NeuP was diagnosed as reported before [50]. The diagnosis of DN was established according to international criteria, based on typical clinical symptoms, nerve conduction studies (NCS) and IENFD determined by skin biopsy and/or abnormal TTT, after excluding all other known causes of neuropathy [51]. The DN patients were divided according to pain sensitivity into the following two groups: painful-DN subpopulation and painless-DN subpopulation. The patients with pain for at least 1 year, who reported a PI-NRS score of ≥ 4 despite analgesic or before starting treatment were defined as painful-DN; those with a pain score of ≤3 were defined as painless-DN.

### 4.2. DNA Isolation and Storage

Genomic DNA was extracted from whole blood using the NucleoSpin8 Blood Isolation Kit (Macherey-Nagel, Düren, Germany) or QIAamp DNA Blood Maxi Kit, Puregene^®^ Blood Core Kit (Qiagen, Hilden, Germany), according to the manufacturer’s instructions at Maastricht UMC+, and IRCCS Neurological Institute Carlo Besta. DNA samples were coded and stored in the central DNA bank at Maastricht UMC+ and IRCCS Neurological Institute Carlo Besta in −20 °C.

### 4.3. Gene Panel and smMIPs-NGS Protocol

Gene candidates for smMIPs-NGS were selected based on their role with pain in OMIM and the literature, high expression in peripheral nerve DRG and/or trigeminus cells, and by comparative and integrative genomics, with SCN9A co-expression described by Szklarczyk et al. [52]. The fifteen ion channel gene panel included the following: anoctamin 1 and 2 (ANO1, ANO3), hyperpolarization-activated cyclic nucleotide-gated potassium channel 1 (HCN1), potassium voltage-gated channel, shaker-related subfamily, member 2 and member 4 (KCNA2, KCNA4), potassium channel, subfamily K, member 18 (KCNK18), potassium intermediate/small conductance calcium-activated channel, subfamily N, member 1 (KCNN1), potassium voltage-gated channel, KQT-like subfamily, member 3 and member 5 (KCNQ3, KCNQ5), potassium voltage-gated channel, delayed-rectifier, subfamily S, member 1 (KCNS1), transient receptor potential cation channel, subfamily A, member 1 (TRPA1), transient receptor potential cation channel, subfamily M, member 8 (TRPM8), transient receptor potential cation channel, subfamily V, member 1, member 3 and member 4 (TRPV1, TRPV3, TRPV4) (Table S1).

In total, 295 single-molecule molecular inversion probes (smMIPs) were designed to capture exon and exon-flanking intron sequences (+/−20 bps) of 15 ion channel gene candidates (Table S1), using a modified version of the MIPgen tool (http://shendurelab.github.io/MIPGEN/, accessed on 2 September 2014). The number smMIPs per gene and size of targeted coding region per gene have been given in Table S3. All probes, synthesized by Integrated DNA Technologies (IDT, Iowa, ID, USA), were 77–80-mers long, containing extension and ligation arm, joined by a common linker with two universal PCR primer sites, as described before [53]. The smMIPs with a high arm copy count (>5×) and/or common nucleotide polymorphisms (SNPs) (>1%) in the extension and ligation arms were excluded. The gap-fill length was 220–230 nt. Each probe contains a 5 nt unique molecular identifier (UMI), which enables the removal of duplicates introduced during PCR amplification and sequencing.

SmMIP-NGS was performed as described before [53]. In brief, 50–100 ng of genomic DNA was used for hybridization. After gap filling and ligation, circularized DNA molecules served as the PCR template. In the PCR reaction, universal primers complementary to the linker were used. Amplified samples were pooled, purified by Ampure XP beads (Beckman Coulter, Inc, Brea, California) and paired-end sequenced (2 × 250 bp) using NextSeq 500 (Illumina, Inc., San Diego, CA, USA).

### 4.4. Data Analysis

Sequenced data were analyzed using an smMIPs-NGS pipeline developed by Maastricht UMC+ in collaboration with Radboud University [53]. The smMIP arms were trimmed by our pipeline and then the sequences were aligned to the human reference sequence GRCh37. Duplicates were removed based on UMI probe sequences. Variant calling was performed using GATK (haplotype caller and unified genotyper) following best practices [54]. Variants were annotated based on information and frequencies from ExAC, dbSNP, cadd and Gencode [55]. Furthermore, the pipeline calculates coverage per sample, number of bases, mean and median coverage per MIP target [53].

### 4.5. Variant Interpretation

Exonic variants and exonic flanking regions (+/−20 bps) with >20× coverage and an alternative variant call of >30% were analyzed in further detail. Common SNPs were excluded from the analysis; only variants with dbSNP < 5%, ExAC < 5% and in house database of 12,244 exomes < 1% were included. Alamut Visual (Interactive Biosoftware, Rouen, France) software was used for variant interpretation. Variants were classified according to the practice guidelines of the Association for Clinical Genetic Science (ACGS) [22]. Potentially pathogenic variants were checked manually, using BAM visualization in the Alamut Visual software and confirmed by Sanger sequencing. Due to the structure of the cohort, co-segregation of potentially pathogenic variants with the disease was not possible.

### 4.6. Statistical Analysis

The independent Student’s *t*-test was used to analyze continuous variables and chi-square test was applied for categorical variables. The significance level was defined as 0.05.

## Figures and Tables

**Table 1:** Potentially pathogenic variants of ion channel genes identified in patients with painful-diabetic neuropathy (*n* = 222).

Gene	c. Position ^&^	p. Position	Number of Patients	Classification Based Richards et al. [22]	Location	MAF GnomAD (%)	Ref.
*ANO3*	c.638C>T	p.(Ser213Phe)	1	VUS	N-terminus	0	-
	c.1357A>G	p.(Ile453Val)	1	VUS	N-terminus	0	-
	c.2950C>T	p.(Leu984Phe)	1	VUS	Transmembrane domain VIII	0.0046	-
*HCN1*	c.1214G>A	p.(Arg405Gln)	1	VUS	C-terminus	0.0004	-
*KCNK18*	c.414_415del	p.(Phe139Trpfs*25)	1	VUS	Exon 3, the new reading frame ends in a STOP codon at position 25	0.043	[19,20,21,23]
	c.361dup	p.(Tyr121Leufs*44)	1	VUS	Exon 3, the new reading frame ends in a STOP codon at position 44	0.024	[19]
*TRPA1*	c.2481del	p.(Ala828Leufs*17)	1	VUS	Exon 21, the new reading frame ends in a STOP codon at position 17	0	-
	c.352C>G	p.(Leu118Val)	1	VUS	Ankyrin repeat II-containing domain	0.047	-
	c.1954C>T	p.(Arg652*)	1	VUS	Cytoplasmic domain between ANK repeats and 1st transmembrane domain	0.015	-
*TRPM8*	c.2114del	p.(Val705Glyfs*79)	1	VUS	Exon 16, the new reading frame ends in a STOP codon at position 79	0.0004	-
	c.1437G>T	p.(Glu479Asp)	1	VUS	N-terminus	0.012	-
	c.2195C>T	p.(Thr732Ile)	1	VUS	Linker between transmembrane domain I and II	0.069	-
*TRPV4*	c.2336+1G>A	p.? ^#^	1	VUS	Donor splice site of intron 14	0.0012	-

c. position, location cDNA; p. position, location in protein; MAF gnomAD, minor allele frequency the Genome Aggregation Database; n/a, not applicable; ^&^ variants detected were annotated according to the guidelines of the Human Genome Variation Society using reference sequence GRCh37 and transcript numbers, NM_001313726.1 (*ANO3)*; NM_021072.4 (*HCN1)*, NM_181840.1 (*KCNK18)*, NM_007332.2 (*TRPA1)*; NM_024080.4 (*TRPM8)*, NM_021625.4 (*TRPV4)*; * one patient was heterozygous for *TRPA1* c.2481del and *TRPM8* c.1437G>T; ^#^ predicted skip of exon 14 would lead to a premature stop codon.

**Table 2:** Clinical characteristic of patients with painful-diabetic neuropathy and ion channel gene variant.

Sex	Age	DM Type	Age of Onset DM	Age of Onset Neuropathy	Max Pain during Night	Mean Pain during Night	Max Pain during Day	Mean Pain during Day	Potential Underlying Cause of Neuropathy	TTT	Positive Family History for Neuropathy	Variant
F	61	DM2	34	54	6	4	5	3	Adenoma of the thyroid gland, hypothyroidism	Abnormal (feet)	Yes	ANO3 p.(Ser213Phe)
F	68	DM2	43	60	6	4	10	4	Unknown	Normal	No	ANO3 p.(Ile453Val)
M	43	DM2	34	37	0	0	8	2	Slipped disk (three times)	Normal	No	ANO3 p.(Leu984Phe)
M	65	DM2	57	65	9	9	4	3	Back surgery after a car crash, slipped disk	Abnormal (feet and hands)	No	HCN1 p.(Arg405Gln)
F	75	DM2	49	72	8	3	7	3	Unknown	Abnormal (feet and hands)	No	KCNK18 p.(Phe139Trpfs*25)
M	77	DM2	60	-	-	-	-	-	-	-	-	KCNK18 p.(Tyr121Leufs*44)
M	48	DM2	33	-	-	-	-	-	-	-	No	TRPA1 p.(Ala828Leufs *17) TRPM8 p.(Glu479Asp)
M	65	DM2	65	65	10	10	8	8	Spine injury	Abnormal (feet)	No	TRPA1 p.(Leu118Val)
M	73	DM2	58	69	8	6	8	6	Unknown	Abnormal (feet)	No	TRPA1 p.(Arg652*)
M	57	DM2	18	46	7	0	7	0	Hypothyroidism	Abnormal (feet and hands)	Yes	TRPA8 p.(Val705Glyfs*79)
F	77	DM2	75	75	9	5	9	6	Hypothyroidism	Abnormal (feet and hands)	No	TRPM8 p.(Thr732Ile)
M	73	DM1	8	-	-	-	-	-	-	-	-	TRPV4 p.?

DM, diabetes mellitus; DM1, diabetes mellitus type 1; DM2, diabetes mellitus type 2; F, female; M, male; -, data incomplete; TTT, thermal threshold testing. Age given at the day of recruitment visit. Neuropathic pain assessed using Pain Intensity Numerical Rating Scale.

**Table 3:** Potentially pathogenic variants of ion channel genes identified in patients with painless-diabetic neuropathy (*n* = 304).

Gene	c. Position ^&^	p. Position	Number of Patients	Classification Based Richards et al. [22]	Location	MAF GnomAD (%)	Ref.
*ANO1*	c.1892G>A	p.(Arg631Gln)	1	VUS	Linker between transmembrane domain V and VI	0.012	-
*KCNK18*	c.414_415del	p.(Phe139Trpfs*25)	1	VUS	Exon 3, the new reading frame ends in a STOP codon at position 25	0.043	[19,20,21,23]
	c.361dup	p.(Tyr121Leufs*44)	2	VUS	Exon 3, the new reading frame ends in a STOP codon at position 44	0.024	[19]
*KCNQ3*	c.1226C>G	p.(Pro409Arg)	1	VUS	C-terminus	0.067	-
*TRPA1*	c.352C>G	p.(Leu118Val)	1	VUS	Ankyrin repeat II-containing domain	0.047	-
	c.1980C>A	p.(Phe660Leu)	1	VUS	Cytoplasmic domain between ANK repeats and transmembrane domain I	0.01	-
*TRPM8*	c.2956G>A	p.(Val986Ile)	1	VUS	C-terminus	0.002	-
*TRPV1*	c.1450G>C	p.(Gly484Arg)	1	VUS	Transmembrane domain II	0	-
	c.1781C>T	p.(Ala594Val)	1	VUS	Transmembrane domain V	0.042	-
	c.1790C>T	p.(Thr597Met)	1	VUS	Transmembrane domain V	0.0021	-
*TRPV4*	c.711A>G	p.? ^#^	1	VUS	Ankyrin repeat I-containing domain	0.0008	-
	c.958C>T	p.(Arg320*)	1	VUS	N-terminus	0.0039	-
	c.1039G>T	p.(Asp347Tyr)	1	VUS	N-terminus	0.018	-

c. position, location cDNA; p. position, location in protein; MAF gnomAD, minor allele frequency the Genome Aggregation Database; n/a, not applicable; VUS, variants with uncertain clinical significance. ^&^ Variants detected were annotated according to the guidelines of the Human Genome Variation Society using reference sequence GRCh37 and transcript numbers, NM_018043.5 (*ANO1*); NM_181840.1 (*KCNK18*), NM_004519.3 (*KCNQ3*), NM_007332.2 (*TRPA1*); NM_024080.4 (*TRPM8*); NM_080706.3 (*TRPV1*)*;* NM_021625.4 (*TRPV4*); ^#^ predicted alternative splice sites would lead to a premature stop codon.

**Table 4:** Clinical characteristic of patients with painless-diabetic neuropathy and ion channel gene variant.

Sex	Age	DM Type	Age of Onset DM	Age of Onset Neuropathy	Potential Underlying Cause of Neuropathy	TTT	Positive Family History for Neuropathy	Variant
M	78	DM2	55	-	-	-	No	ANO1 p.(Arg631Gln)
M	68	DM2	64	-	-	-	-	KCNK18 p.(Phe139Trpfs*25)
M	50	DM2	46	-	-	-	No	KCNK18 p.(Tyr121Leufs*44)
M	73	DM2	57	-	-	-	-	KCNK18 p.(Tyr121Leufs*44)
M	69	DM1	13	-	-	-	-	KCNQ3 p.(Pro409Arg)
F	61	DM1	32	61	Thyroidectomy	Abnormal (feet)	Yes	TRPA1 p.(Leu118Val)
F	39	DM2	26	-	-	-	-	TRPA1 p.(Phe660Leu)
M	81	DM2	66	78	Hypothyroidism	Abnormal (hands and feet)	No	TRPM8 p.(Val986Ile)
M	54	DM1	7	-	-	-	-	TRPV1 p.(Gly484Arg)
F	62	DM2	60	-	-	-	-	TRPV1 p.(Ala594Val)
M	43	DM1	17	35	Unknown	Abnormal (hands and feet)	Yes	TRPV1 p.(Thr597Met)
M	76	DM1	23	66	Slipped disc LS 5/S1	Normal	No	TRPV4 p.?
M	52	DM2	50	-	-	-	-	TRPV4 p.(Arg320*)
M	67	DM2	67	68	-	Normal	No	TRPV4 p.(Asp347Tyr)

DM, diabetes mellitus; DM1, diabetes mellitus type 1; DM2, diabetes mellitus type 2; F, female; M, male; -, data incomplete. Age given at the day of recruitment visit.

## Data Availability

Not applicable.

## Supplementary Materials

The following supporting information can be downloaded at: https://www.mdpi.com/article/10.3390/ijms23137190/s1.

## Disclaimer

Professor Stephen Waxman received SGW reports grants from European Union’s Horizon 2020 research and innovation programme Marie Sklodowska-Curie grant for PAIN-Net, Molecule-to-Man Pain Network (grant no. 721841) and European Union 7th Framework Programme (grant no. 602273) for the PROPANE study, was supported in part by the Rehabilitation Research and Development Service and Biomedical Laboratory Research Service, Department of Veterans Affairs, has served as a consultant to ChromoCell Corp. and Sangamo Therapeutics, and has served on the Scientific Advisory Board of OliPass Corporation, MedTronics and Navega Therapeutics, outside the submitted work.
