# Supplementary material for: Peripheral Ion Channel Gene Screening in Painful- and Painless-Diabetic Neuropathy
Source: Int J Mol Sci. 2022 Jun 28;23(13):7190. doi: 10.3390/ijms23137190 (PMC9266298; doi:10.3390/ijms23137190)
Supplement: Supplementary file 1 [file ijms-23-07190-s001.zip › ijms-1774358-supplementary.pdf]

Supplementary materials

Table S1. Peripheral ion channels panel

**Gene**	**OMIM number**	**Full gene name**
ANO1	610108	Anoctamin 1, calcium activated chloride channel
ANO3	610110	Anoctamin 3
HCN1	602780	Hyperpolarization activated cyclic nucleotide-gated potassium channel 1
KCNA2	176262	Potassium voltage-gated channel, shaker-related subfamily, member 2
KCNA4	176266	Potassium voltage-gated channel, shaker-related subfamily, member 4
KCNK18	613655	Potassium channel, subfamily K, member 18
KCNN1	602982	Potassium intermediate/small conductance calcium-activated channel, subfamily N, member 1
KCNQ3	602232	Potassium voltage-gated channel, KQT-like subfamily, member 3
KCNQ5	607357	Potassium voltage-gated channel, KQT-like subfamily, member 5
KCNS1	602905	Potassium voltage-gated channel, delayed-rectifier, subfamily S, member 1
TRPA1	604775	Transient receptor potential cation channel, subfamily A, member 1
TRPM8	606678	Transient receptor potential cation channel, subfamily M, member 8
TRPV1	602076	Transient receptor potential cation channel, subfamily V, member 1
TRPV3	607066	Transient receptor potential cation channel, subfamily V, member 3
TRPV4	605427	Transient receptor potential cation channel, subfamily V, member 4

Table S2. Characteristics of patients with painful- and painless- Diabetic Neuropathy

	Painful-DN	Painless-DN	Total	p
Mean age at recruitment [years +/- SD]	64.2 (+/- 10.3)	64.8 (+/- 11.9)	64.5 (+/- 11.2)	0.56*
Females (n, %)	82 (36.9)	72 (23.7)	154 (29.3)	< 0.0001&
Males (n, %)	140 (63.1)	232 (76.3)	372 (70.7)	
DM1 (n, %)	34 (30.4)	78 (69.4)	112 (21.3)	0.0032&
DM2 (n, %)	190 (45.9)	224 (54.1)	414 (78.7)	
Mean age of onset DM1 [years +/- SD]	25.9 (+/- 15.2)	27.8 (+/- 16.1)	27.2 (+/- 11.2)	0.56*
Mean age of onset DM2 [years +/- SD]	52.1 (+/- 12.1)	55.1 (+/- 10.9)	53.7 (+/- 11.6)	0.007*
Mean age of onset neuropathy [years +/- SD]	59.1 (+/-11.1)	62.3 (+/-11.7)	60.8 (+/-11.5)	0.012*
Duration of neuropathy [years +/- SD]	6.9 (+/- 5.9)	5.5 (+/-6.5)	6.2 (+/- 6.2)	0.25*
Positive family history for neuropathy (n, %)	30 (18.6)	159 (56.8)	189 (42.9)	< 0.0001&
Negative family history for neuropathy (n, %)	131 (81.4)	121 (43.2)	252 (57.1)	
Max pain during night [PI-NRS]	6.2 (+/- 3.0)	0.5 (+/- 1.5)	3.2 (+/- 3.7)	< 0.0001*
Mean pain during night [PI-NRS]	3.7 (+/- 2.8)	0.2 (+/- 0.8)	1.8 (+/- 2.7)	< 0.0001*
Max pain during day [PI-NRS]	5.8 (+/- 3.0)	0.6 (+/-1.6)	3.1 (+/- 3.5)	< 0.0001*
Mean pain during day [PI-NRS]	3.4 (+/-2.6)	0.2 (+/- 0.8)	1.7 (+/- 2.5)	< 0.0001*
Normal TTT (n, %)	32 (20.1)	44 (24.3)	76 (22.4)	0.36&
Abnormal TTT (n, %)	127 (79.9)	137 (75.7)	264 (77.6)	

*Independent Student's t-test, &chi-square test, significance level <0.05

Table S3. Average coverage of MIP-NGS of 15 ICG

Gene name	Number of MIP [n]	Targeted coding region [bp]	Total number of nucleotides not covered by MIP <20x/bp [bp]	Location of missing area	Average coverage >30x/bp [%]
ANO1	32	2961	441	ex1*, ex2, ex12*, ex20*, ex26	85.1
ANO3	30	2946	0	-	100
HCN1	18	2673	425	ex1*	84.1
KCNA2	10	1500	0	-	100
KCNA4	10	1962	0	-	100
KCNK18	7	1155	0	-	100
KCNN1	14	1632	249	ex6, ex10, ex11	84.7
KCNQ3	21	2619	256	ex1	90.2
KCNQ5	23	2856	192	ex1	93.3
KCNS1	9	1581	181	ex4	88.6
TRPA1	31	3360	111	ex1	96.7
TRPM8	32	3315	0	-	100
TRPV1	20	2520	153	ex3*	93.9
TRPV3	19	2376	348	ex2, ex3, ex7	85.4
TRPV4	19	2616	214	ex3, ex9	91.8

bp, base pair, ex, exon, n, number

*large part of exon <20x coverage/bp or sequence data is missing completely

Table S4. Mean pain score in patients with painful- Diabetic Neuropathic with and without an ion channel variant

	Max pain during night	Mean pain during night	Max pain during day	Mean pain during day
patients with ICG variant (n=9) [+/- SD]	7.00 [+/- 2.96]	4.56 [+/- 3.47]	7.33 [+/-1.87]	3.89 [+/-2.42]
patients without ICG variant (n=150) [+/- SD]	6.2 [+/-3.0]	3.7 [+/-2.8]	5.8 [+/-3.0]	3.4 [+/-2.6]

Pain intensity was evaluated using numerical rating scale PI-NRS.

Table S5. Autonomic complaints reported by patients with painful- Diabetic Neuropathy (n=9) carrying variant in ICG.

**Variant**	**sweating change**	**diarrhea**	**constipation**	**micturition problems**	**dry eyes**	**dry mouth**	**orthostatic dizziness**	**Palpitations**	**hot flashes**	**hypersensitivity of leg's skin**	**burning feet**	**sheet intolerance**	**restless leg**
ANO3 p.(Ser213Phe)	1	2	0	0	1	0	2	3	1	3	3	0	1
ANO3 p.(Ile453Val)	1	0	0	0	3	0	1	1	1	3	2	3	1
ANO3 p.(Leu984Phe)	3	1	1	0	2	2	1	0	0	2	1	0	0
HCN1 p.(Arg405Gln)	2	1	1	0	2	2	1	2	0	2	2	2	1
KCNK18 p.(Phe139Trpfs*25)	1	1	1	1	0	2	2	0	0	2	1	1	0
TRPA1 p.(Leu118Val)	1	1	1	0	0	1	1	0	0	1	2	3	3
TRPA1 p.(Arg652*)	0	1	1	2	2	2	1	1	0	1	2	1	0
TRPA1 p.(Val705Glyfs*79)	0	1	0	0	0	1	1	0	0	1	1	0	0
TRPM8 p.(Thr732Ile)	2	0	2	0	2	2	1	1	1	1	1	0	1

Numeric scale (1-4) expresses frequency of complaints; 0, never; 1, sometimes; 2, often; 3, always; -, not determined. Three painful-DN patients not shown in the table, data incomplete

Table S6. Autonomic complaints reported by patients with painless- Diabetic Neuropathy (n=4) carrying a variant in ICG.

Variant	sweating change	diarrhea	constipation	micturition problems	dry eyes	dry mouth	orthostatic dizziness	Palpitations	hot flashes	hypersensitivity of leg's skin	burning feet	sheet intolerance	restless leg
TRPA1 p.(Leu118Val)	1	0	1	1	3	1	0	1	0	2	0	0	0
TRPM8 p.(Val986Ile)	1	0	1	2	1	0	0	1	1	1	1	0	0
TRPV4 p.(Thr597Met)	0	1	0	0	0	0	1	1	0	1	0	0	0
TRPV4 p.?	2	0	1	2	3	1	1	1	0	0	1	0	0

Numeric scale (1-4) expresses frequency of complaints; 0, never; 1, sometimes; 2, often; 3, always; -, not determined. Data presented only for patients with ICG that fully completed the autonomic complaints questionnaire
